# Supplementary material for: Reductions in prefrontal activation predict off-topic utterances during speech production
Source: Nat Commun. 2019 Jan 31;10:515. doi: 10.1038/s41467-019-08519-0 (PMC6355898; doi:10.1038/s41467-019-08519-0)
Supplement: Supplementary file 1 — Supplementary Information [file 41467_2019_8519_MOESM1_ESM.pdf]

Hoffman. Reductions in prefrontal activation predict off-topic utterances during speech production

SUPPLEMENTARY MATERIALS

Supplementary Methods

List of prompts used to elicit speech:

Which is your favourite season and why?

What do you like or dislike about Christmas?

Why do people come to Scotland on holiday?

What sort of things usually happen at a wedding?

Describe a typical visit to a restaurant

What sort of things do you have to do to look after a dog?

What do people usually do when getting ready for work in the morning?

Describe the steps you would take if going somewhere by train

Describe how you would make a cup of tea or coffee

How would you prepare to go on holiday?

What would it have been like to live in the Middle Ages?

What would it be like to live in Antarctica?

What do the police do when a crime has been committed?

What happens when a storm is forecast in the UK?

What are the advantages and disadvantages of going to university?

What sort of things does the Queen do on a typical day?

What happens during a general election in the UK?

Why are some people concerned about climate change?

Do you think it's a good idea to send people to live on Mars?

Do you think the internet has improved people's lives?

Further methods of LSA space and averaging method: The LSA vector space was generated using the British National Corpus [1]. Each document in the BNC was divided into contexts of 1000 words in length. A term-by-context matrix was generated, log-entropy weighting applied and singular value decomposition used to reduce the dimensionality of the matrix to 300 dimensions. These steps were performed using the Text to Matrix Generator toolbox in Matlab [2] and resulted in the creation of latent semantic vectors for 53,758 words. To generate semantic representations for speech windows or whole responses, the vectors for individual words were combined in the following way:

1. A list of words appearing in the speech window was created. Common function words that carry little semantic information were removed from the list.
2. Vectors for each word in the list were retrieved and normalised so that each had a magnitude of one.
3. The vector for each word was weighted according to (a) the log of its frequency in the speech passage being analysed (assigning greater weight to words occurring more frequently in the window) and (b) its entropy value in the BNC (assigning greater weight to words whose presence is more informative about the semantic content of the window).
4. The weighted vectors were averaged to give a single vector representing the semantic content of the words in the window.

These procedures were similar to those used by other researchers [3,4].

Measurement of other characteristics of speech: Transcripts were submitted to the Stanford Log-linear Part-of-Speech Tagger v3.8 for automated part-of-speech tagging [5]. In addition to the coherence measures described in the main text, the following measures were computed for each 5s block of speech:

Number of words: the total number of words produced in the 5s speech block.

Proportion closed-class words: The proportion of words in each block whose part-of-speech was classified as closed-class. Closed-class words included pronouns, numbers, prepositions, conjunctions, determiners, auxiliaries and some adverbs.

Type: token ratio (TTR): This is the ratio of unique lexical items (types) produced to total words (tokens) spoken. A higher value indicates greater lexical diversity. This is the only speech marker that could not be calculated for individual blocks; it was instead calculated for each 50s response as a whole. Because TTR is highly dependent on response length, a moving window approach was adopted to control for the number of words included in the analysis [6]. For each response, the first 50 words were first considered and a TTR calculated over these. The window was then moved forward one word and a new TTR calculated, and this process was repeated until the end of the response was reached. A mean TTR was then computed across all windows.

Mean noun frequency: Frequencies in the SUBTLEX-UK database [7] were obtained for all words tagged as nouns and an average calculated for each block.

Mean noun concreteness: Concreteness ratings for nouns were obtained from Brysbaert et al. [8].

Mean noun age of acquisition (AoA): Estimates of AoA for nouns were obtained from the norms of Kuperman et al. [9].

Mean noun semantic diversity (SemD): SemD values for nouns were obtained from Hoffman et al. [1]. SemD is a measure of variability in the contextual usage of words. Words with high SemD values are used in a wide variety of contexts and thus more variable and less well-specified meanings.

Mean noun number of phonemes: The length of all nouns (in phonemes) was also calculated.

Local coherence: Local coherence refers to the degree to which adjoining utterances in speech are meaningfully related to one another. A measure of local coherence was computed using the same methods as Hoffman et al. [10]. Like the main global coherence metric, this was based on LSA analysis of speech produced by each participant. However, rather than comparing each window of speech to the prototypical response to the topic, each 20-word window was compared to the participant's own previous window. Thus, the measure indexed the degree to which current speech production was meaningfully related to utterances produced earlier in the response.

In cases where a particular measure could not be computed for a block (for example, blocks in which no nouns were produced) the mean value for the rest of the response was used.

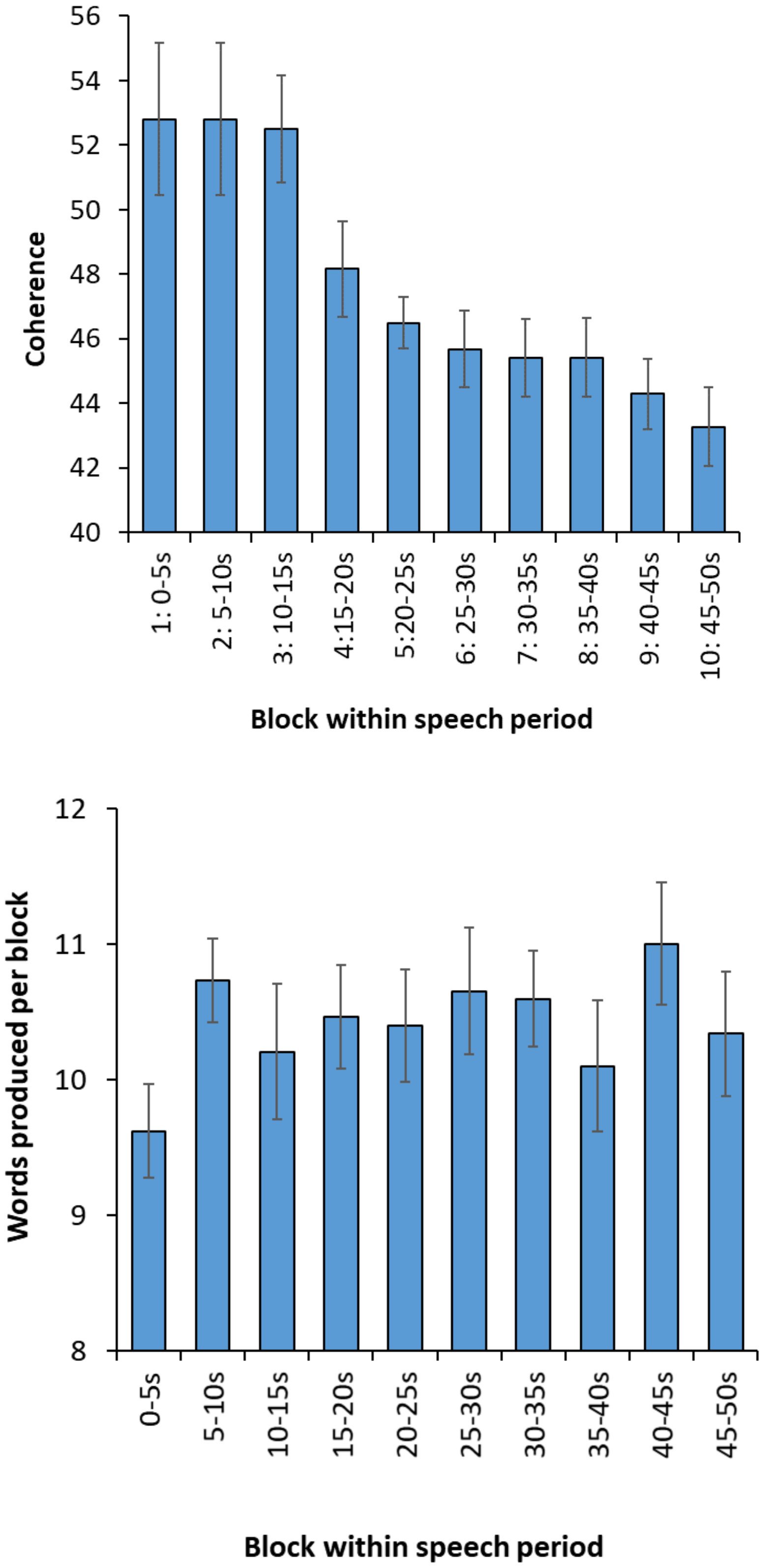

Supplementary Figure 1: Average coherence of speech and number of words produced in each 5s block of the speech production periods. Bars indicate standard error of the mean.

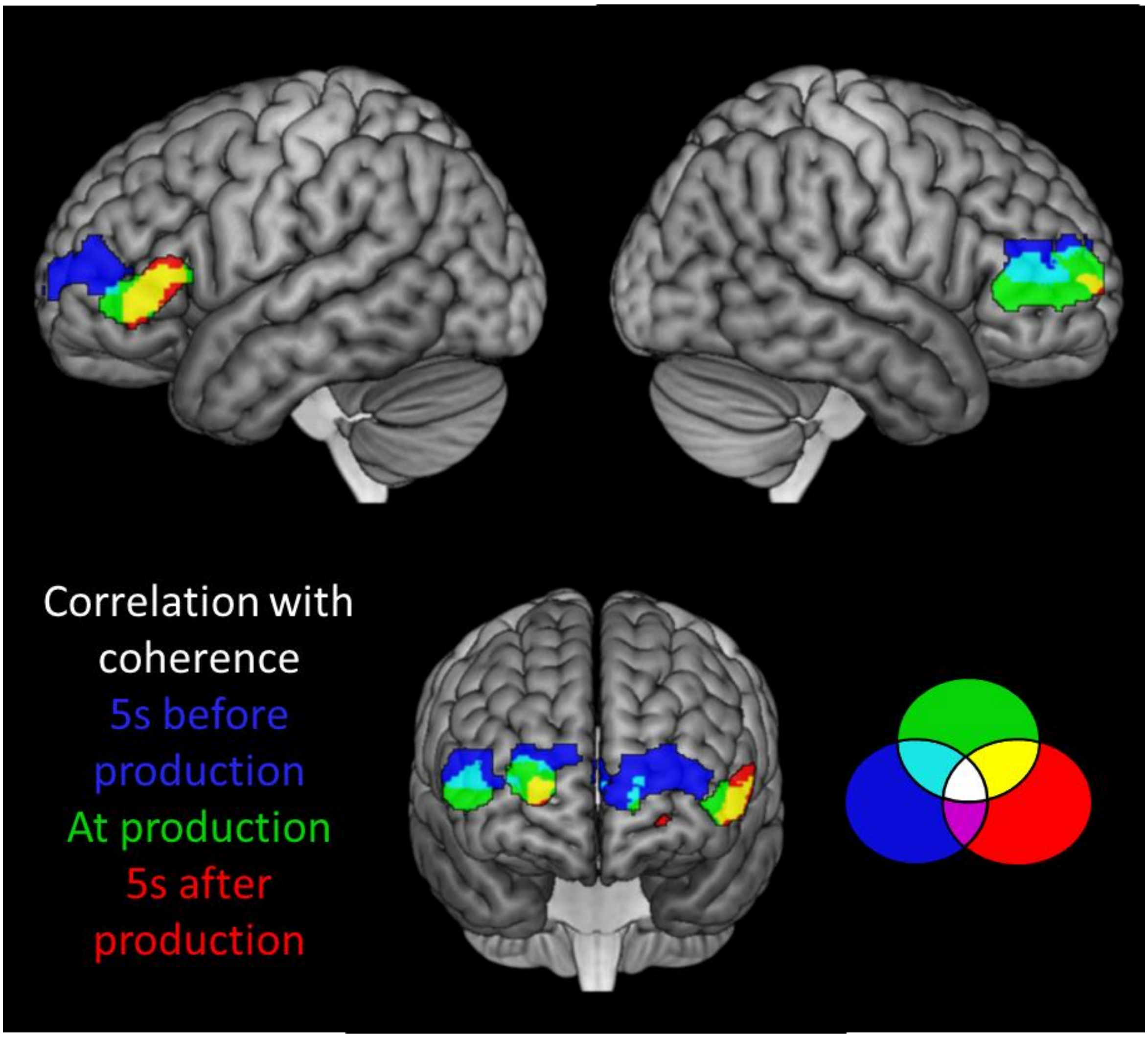

Supplementary Figure 2: Regions showing greater activation when participants produced more coherent speech at time of production (green; standard model), 5s before the speech was produced (blue; early model) and 5s after the speech was produced (red; late model).

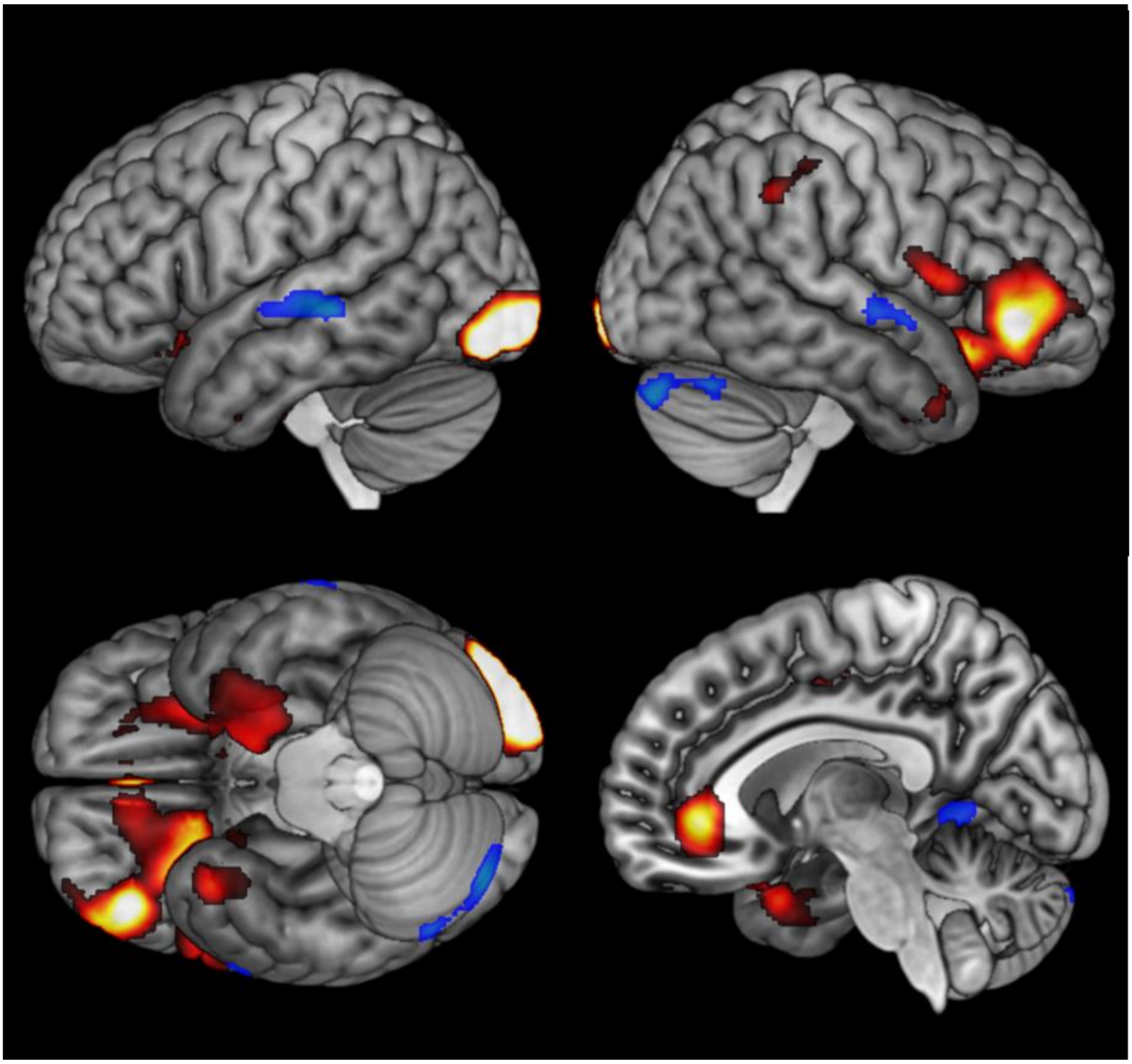

Supplementary Figure 3: Regions showing increasing (hot colours) or decreasing (cool colours) activation over time within speech production periods.

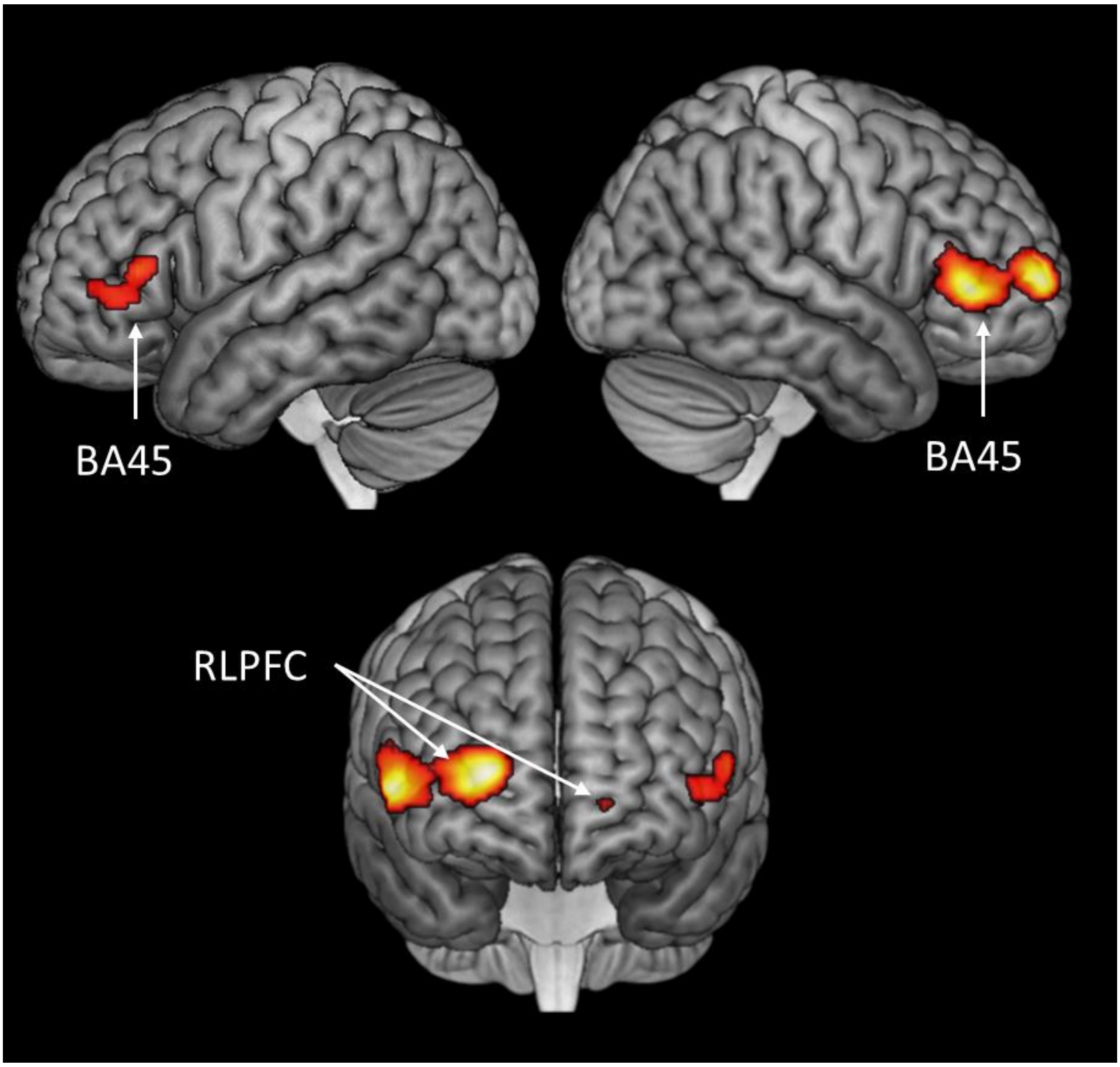

<u>*Supplementary Figure 4: Regions showing a positive correlation with the coherence factor obtained from principal components analysis of speech properties.*</u> *The other PCA factors (corresponding to vocabulary, specificity and verbosity) were entered as covariates in this analysis, thus observed effects cannot be due to covariance with these aspects of speech.*

Supplementary Table 1: Activation peaks for speech planning and production, relative to baseline

Contrast	Location	Extent (mm^3)	*t*	MNI co-ords x	y	z
Speech planning	Occipital pole	95392	10.45	2	-94	-4
	R lingual gyrus		6.87	16	-78	-14
	R cerebellum		6.01	42	-66	-26
	L superior parietal		4.45	-24	-78	54
	L angular gyrus		4.27	-40	-82	38
	R temporal pole	3544	7.41	58	12	-24
	R anterior STS		4.67	68	-2	-10
	L anterior MTG	11256	6.76	-68	-8	-14
	L anterior MTG		6.53	-54	0	-26
	L mid MTG		3.62	-56	-34	-6
	L pars orbitalis	23544	5.83	-44	34	-14
	L middle frontal gyrus		5.56	-44	6	58
	L pars triangularis		5.43	-46	24	4
	Substantia nigra	11336	5.51	-8	-22	-14
	R globus pallidus		5.36	16	2	-4
	L globus pallidus		5.26	-16	4	6
	L SMA	1264	4.03	-6	10	58
Speech production	R cerebellum	22112	8.99	36	-78	-34
	R cerebellum		5.18	20	-92	-30
	R cerebellum		4.83	16	-78	-24
	L lateral occipital	7192	6.60	-44	-84	32
	L angular gyrus		4.20	-42	-64	18
	L superior frontal gyrus	7760	5.26	-8	32	62
	L SMA		4.99	-8	18	60
	L superior frontal gyrus		3.70	-10	50	48
	L middle frontal gyrus	6800	5.24	-40	14	58
	L middle frontal gyrus		5.15	-36	10	64
	L pars triangularis	2376	4.99	-52	24	6
	L anterior MTG	7000	4.91	-60	-8	-28
	L anterior ITG		4.55	-44	-8	-50
	L anterior ITG		4.27	-46	-8	-40

Supplementary Table 2: Activation peaks associated with coherence and time during speech production

Effect	Location	Extent (mm^3)	*t*	MNI co-ords x	y	z
Positive effect of coherence	R frontopolar cortex	11720	7.20	22	58	6
	R pars triangularis		5.49	42	30	4
	R anterior MFG		5.13	48	56	2
	L anterior cingulate	11656	5.23	-16	40	10
	L pars triangularis		4.86	-44	40	-2
	L anterior insula		4.60	-24	30	6
Negative effect of coherence	No peaks					
Increase over time	L lateral occipital	10944	8.27	-42	-90	-16
	L occipital pole		7.41	-26	-104	-6
	R pars orbitalis	55048	5.69	44	38	-6
	Ventromedial PFC		4.91	4	38	-6
	R pars orbitalis		4.63	30	22	-16
	R temporal pole		3.96	36	10	-32
	L parahippocampal gyrus		3.90	-14	-4	-30
	R middle frontal gyrus	3680	5.09	28	6	32
	R precentral gyrus		4.52	34	0	30
	R precentral gyrus		3.70	26	-6	40
	R angular gyrus	1040	3.60	60	-46	40
	R supramarginal gyrus		3.22	58	-34	48
Decrease over time	L posterior STS	3264	4.04	-58	-32	-2
	R cerebellum	2136	4.03	32	-86	-30
	R cerebellum		3.84	50	-66	-28
	R superior temporal gyrus	1040	3.63	66	2	-6
	R superior temporal gyrus		3.52	58	-8	-2
	R lingual gyrus	1360	3.40	14	-54	-2
	R lingual gyrus		3.35	4	-48	0
	Posterior cingulate		3.07	-4	-46	2

Supplementary Table 3: Correlations among properties of speech

	1.	2.	3.	4.	5.	6.	7.	8.	9.
1. Global coherence	--								
2. Number of words	-.01†	--							
3. Type:token ratio	-.10	-.04	--						
4. % closed class	-.05	.24	-.10	--					
5. Noun frequency	-.10	.12	.07	-.03†	--				
6. Noun concreteness	.16	.003†	-.12	-.02†	-12	--			
7. Noun age of acquisition	-.07	-.12	.04	.002†	-.49	-.41	--		
8. Noun semantic diversity	-.18	.06	.09	-.02†	.63	-.40	-.15	--	
9. Noun phoneme length	-.05	-.18	.03†	-.03†	-.42	-.19	.50	-.15	--
10. Local coherence	.39	.04	-.18	-.007†	-.10	.14	-.06	-.12	-.05

Speech measures were computed for each 5s block of speech produced in the experiment (N=3000). Due to the large number of observations, most correlation coefficients were significant at $p<0.05$. † indicates coefficients that did *not* reach this threshold.

Supplementary Table 4: Results of principal components analysis of speech properties

	Factor 1	Factor 2	Factor 3	Factor 4
1. Global coherence	-.03	-.03	**.78**	-.12
2. Number of words	-.17	.07	.02	**.71**
3. Type:token ratio	.01	.09	**-.39**	**-.32**
4. % closed class	.06	-.05	.02	**.80**
5. Noun frequency	**-.58**	**.63**	-.03	-.03
6. Noun concreteness	**-.56**	**.77**	-.03	-.06
7. Noun age of acquisition	**.89**	.08	.00	.02
8. Noun semantic diversity	-.14	**.87**	.00	-.02
9. Noun phoneme length	**.75**	-.06	-.03	-.11
10. Local coherence	.01	.06	**.84**	.01

Table shows pattern matrix following promax rotation. Loadings with absolute values greater than 0.3 have been highlighted in bold.

Supplementary Table 5: Peak co-ordinates for analysis of individual differences in coherence

Effect	Location	Extent (mm^3)	*t*	MNI co-ords x	y	z
Greater activation in more coherent speakers during Planning	R pars triangularis	5433	8.51	54	34	2
	R pars orbitalis		4.82	40	40	-6
Greater activation in less coherent speakers during Planning	L anterior ITG	1360	4.67	-52	-6	-38
	L anterior ITG		3.26	-52	-14	-42
Greater activation in more coherent speakers during Production	L temporal pole	2192	5.93	-38	18	-46
	L anterior ITG		3.39	-48	-2	-50
Greater activation in less coherent speakers during Production	No peaks					

Supplementary Table 6: Demographic information and mean test scores

Measure	Score
Age	77.5 (8.4)
Sex M:F	6:9
Years of education	15.0 (2.9)
MMSE /30	29.5 (0.7)
Category fluency (items per category)	22.3 (3.9)
Letter fluency (items per category)	17.2 (6.7)
Trails A errors	0.1 (0.4)
Trails B errors	1.0 (1.7)
Trails A time (s)	38.5 (13.6)
Trails B time (s)	80.2 (40.4)
SCOLP lexical decision (% correct; chance = 50%)	94.2 (5.2)
Synonym judgement (% correct; chance = 25%)	72.6 (15.2)

Standard deviations are shown in parentheses. MMSE = Mini-mental state examination [11]. SCOLP = Speed and Capacity of Language Processing test [12]. For the MMSE, Trails and SCOLP tests, participants' scores were compared with published normative data [13-15]. All participants scored above the 10th percentile for their sex, age and education level on all tests.

Supplementary Table 7: Examples of highly coherent and less coherent responses to the prompt: What do people usually do when getting ready for work in the morning?

Participant	Block	Speech	Global Coherence
Participant A	1	When people get ready to go to work in the morning, you would	43
	2	normally have set an alarm clock to go off at a time that would … them in enough	43
	3	time to get ready. They would have time for	63
	4	a shower, perhaps a shave, teeth cleaning,	70
	5	hair drying, breakfast. Making the breakfast	69
	6	first of all, of course, hopefully with toast or scrambled eggs or	50
	7	something to start off the day, and a cup of tea or coffee	50
	8	just to settle you down, ready for the day. Many people have to travel	33
	9	to work so they would have to have appropriate time to do so, whether by train	18
	10	coach, car, bicycle or walking	48
Participant B	1	Well it's a matter of getting up, getting out	77
	2	of bed and getting dressed. Washing, shaving, toiletry.	77
	3	Downstairs, breakfast and then	80
	4	make plans for the day, like going to work.	45
	5	Because being aged ninety two and having	5
	6	retired twenty eight years ago, it's a long time since I was personally….	10
	7	I very much enjoy the life I have	15
	8	without the demands of the call or the urgency	11
	9	of going through to a work situation. I certainly	19
	10	enjoy not having to drive and finish the morning	53

Supplementary References

1. Hoffman, P., Lambon Ralph, M. A. & Rogers, T. T. Semantic diversity: A measure of semantic ambiguity based on variability in the contextual usage of words. *Behavior Research Methods* **45**, 718-730 (2013).
2. Zeimpekis, D. & Gallopoulos, E. in *Grouping Multidimensional Data* (eds J. Kogan, C. Nicholas, & M. Teboulle) 187-210 (Springer, 2006).
3. Foltz, P. W. Discourse coherence and LSA. *Handbook of latent semantic analysis*, 167-184 (2007).
4. McNamara, D. S., Cai, Z. & Louwerse, M. M. in *Handbook of latent semantic analysis* 379-400 (2007).
5. Toutanova, K., Klein, D., Manning, C. D. & Singer, Y. in *Proceedings of the 2003 Conference of the North American Chapter of the Association for Computational Linguistics on Human Language Technology-Volume 1.* 173-180 (Association for Computational Linguistics).
6. Covington, M. A. & McFall, J. D. Cutting the Gordian knot: The moving-average type–token ratio (MATTR). *Journal of quantitative linguistics* **17**, 94-100 (2010).
7. Van Heuven, W. J., Mandera, P., Keuleers, E. & Brysbaert, M. SUBTLEX-UK: A new and improved word frequency database for British English. *The Quarterly Journal of Experimental Psychology* **67**, 1176-1190 (2014).
8. Brysbaert, M., Warriner, A. B. & Kuperman, V. Concreteness ratings for 40 thousand generally known English word lemmas. *Behavior research methods* **46**, 904-911 (2014).
9. Kuperman, V., Stadthagen-Gonzalez, H. & Brysbaert, M. Age-of-acquisition ratings for 30,000 English words. *Behavior Research Methods* **44**, 978-990 (2012).
10. Hoffman, P., Loginova, E. & Russell, A. Poor coherence in older people's speech is explained by impaired semantic and executive processes. *eLife* **7**, e38907, doi:10.7554/eLife.38907 (2018).
11. Folstein, M. F., Folstein, S. E. & McHugh, P. R. Mini-mental state: A practical method for grading the cognitive state of patients for the clinician. *Journal of Psychiatric Research* **12**, 189-198 (1975).
12. Baddeley, A., Emslie, H. & Smith, I. N. *The Speed and Capacity of Language Processing test*. (Thames Valley Test Company, 1992).
13. Tombaugh, T. Trail Making Test A and B: Normative data stratified by age and education. *Archives of Clinical Neuropsychology* **19**, 203-214, doi:10.1016/s0887-6177(03)00039-8 (2004).
14. Crowell, T. A., Vanderploeg, R. D., Small, B. J., Graves, A. B. & Mortimer, J. A. Elderly norms for the Spot-the-Word test. *Archives of clinical neuropsychology* **17**, 123-130 (2002).
15. Huppert, F. A., Cabelli, S. T. & Matthews, F. E. Brief cognitive assessment in a UK population sample–distributional properties and the relationship between the MMSE and an extended mental state examination. *BMC geriatrics* **5**, 7 (2005).
